# Fatigue Life Assessment of Filled Rubber by Hysteresis Induced Self-Heating Temperature

**DOI:** 10.3390/polym12040846

**Published:** 2020-04-07

**Authors:** Wenbo Luo, Youjian Huang, Boyuan Yin, Xia Jiang, Xiaoling Hu

**Affiliations:** 1 Hunan Key Laboratory of Geomechanics and Engineering Safety, Xiangtan University, Xiangtan 411105, China; 2 College of Civil Engineering and Mechanics, Xiangtan University, Xiangtan 411105, China; byhyj@21cn.com (Y.H.); jiangxia127@163.com (X.J.); 3 Zhuzhou Times New Materials Technology Co. Ltd., Zhuzhou 412001, China; 4 School of Civil Engineering, Hunan University of Science and Technology, Xiangtan 411201, China; yinboyuanxtu@163.com

**Keywords:** fatigue life, filled rubber, hysteresis loss, temperature increase, S–N curve

## Abstract

As a viscohyperelastic material, filled rubber is widely used as a damping element in mechanical engineering and vehicle engineering. Academic and industrial researchers commonly need to evaluate the fatigue life of these rubber components under cyclic load, quickly and efficiently. The currently used method for fatigue life evaluation is based on the S–N curve, which requires very long and costly fatigue tests. In this paper, fatigue-to-failure experiments were conducted using an hourglass rubber specimen; during testing, the surface temperature of the specimen was measured with a thermal imaging camera. Due to the hysteresis loss during cyclic deformation, the temperature of the material was found to first rise and then level off to a steady state temperature, and then it rose sharply again as failure approached. The S–N curve in the traditional sense was experimentally determined using the maximum principal strain as the fatigue parameter, and a relationship between the steady state temperature increase and the maximum principal strain was then established. Consequently, the steady state temperature increase was connected with the fatigue life. A couple of thousand cycles was sufficient for the temperature to reach its steady state value during fatigue testing, which was less than one tenth of the fatigue life, so the fatigue life of the rubber component could be efficiently assessed by the steady state temperature increase.

## 1. Introduction

Because of their hyperelasticity and the energy damping behavior of elastomeric materials, rubber damping elements, such as V-springs, tapered springs, air springs, track dampers, engine mounting pads, joint bushings, etc., are widely used in mechanical engineering, aerospace engineering and vehicle engineering [1]. Due to the diversity of their structural forms, complex viscohyperelasticity of the rubber material and significant physical and geometric nonlinear behaviors under external forces, it is very difficult to predict the fatigue life of these rubber dampers.

Currently, for practical evaluation, fatigue failure tests are carried out on structurally similar prototypes or real structures by applying the designated load spectrum to obtain the S–N curve, which relates the fatigue life with a certain mechanical quantity. The mechanical quantities concerned are usually based on stress (e.g., the maximum stress or stress amplitude) [2,3]; strain (e.g., the maximum strain or strain amplitude) [4,5]; or energy (e.g., the strain energy release rate and the cracking energy density, etc.) [6,7,8]. For testing convenience, the S–N curve is usually established by mechanical parameters based on stress or strain. Obviously, the fatigue life data are only valid for the specific structures tested and are not applicable to other structures. As a consequence, testing requires a considerable time and number of specimens. To reduce this cost, it is necessary to find alternative fatigue control parameters rather than stress- or strain-based quantities. In recent years, rapid estimation methods for mean fatigue limits of metallic materials have been developed based on temperature measurements [9]. Some recent publications [10,11,12,13] proved that the characterization of the fatigue properties of fiber-reinforced thermoplastics can be very much accelerated by the use of the “heat build-up” approach. Le Saux and Marco, et al. [14] tried to use this method for rubber-like materials. They linked the temperature rise to the principal maximum strain, and discussed the relationship between the thermal measurements and the fatigue properties of 15 industrial materials. However, there are few models that bridge the heat build-up with the fatigue life of rubber materials. 

The stress–strain curve of rubber materials under cyclic loading shows a hysteresis loop due to viscoelasticity, and the area of the loop represents the energy loss per unit volume in a deformation cycle. The loss of energy eventually dissipates into heat. When the heat is not allowed to flow out to the environment in time, the temperature of the material rises [15,16,17,18], showing a sharp increase as failure approaches. Such a sudden rise in temperature can be regarded as the precursor to fatigue failure. Therefore, the objective of this paper is to correlate the fatigue life with the self-heating temperature increase, and to develop an efficient method for evaluating the fatigue life of rubber structures.

## 2. Experiments

### 2.1. Materials and Specimens 

The rubber material used for the tests was provided by Zhuzhou Times New Material Technology Co., Ltd. in Zhuzhou, China. The formulation of the rubber compounds was as follows: 100 phr Thailand RSS3 natural rubber, 20 phr N550 carbon black, 10 phr zinc oxide, 5 phr antioxidant, 2.5 phr sulfur, 2 phr stearic acid, 2 phr wax, 2 phr solid coumarone resin, 1.4 phr vulcanization activator. Hourglass rubber specimens, 24 mm long and with a minimum diameter of 14.6 mm, were used in the fatigue failure tests.

### 2.2. Fatigue Tests and Temperature Measurements

**Figure 1:**
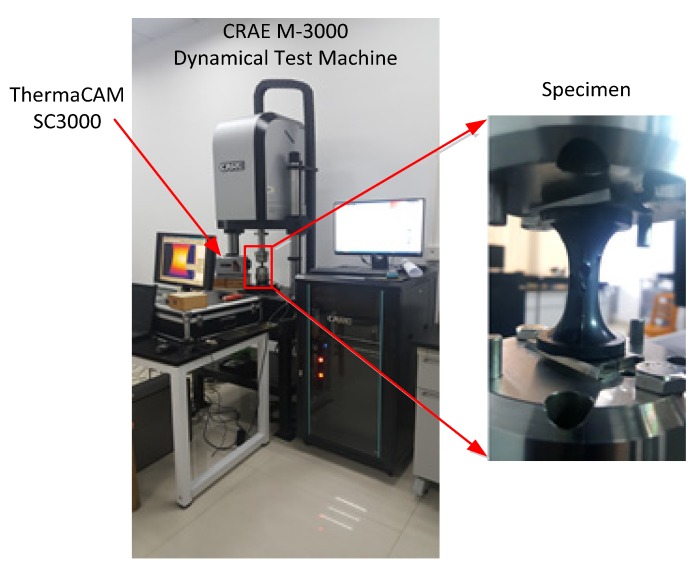
Test setup for fatigue and infrared thermal imaging measurement.

The fatigue-to-failure experiments were conducted on the hourglass specimens using an electromagnetic dynamic testing machine (CARE M-3000, CARE Measurement & Control Co. Ltd., Tianjin, China) in force control mode at room temperature. The sinusoidally varying loads were applied to the specimens at a frequency of 5 Hz and with a load ratio of 0; that is, the minimum load was fixed to be 0N and the maximum load varied from 250 N to 400 N in the four independent fatigue tests. The cycles to failure were recorded for each specimen. Duplicate tests were conducted for each loading case. In order to quantify the hysteresis dissipation, the surface temperature of the specimen was measured with a FLIR ThermaCAM SC3000 thermal imaging camera (FLIR Systems Inc., Orlando, FL, USA) during the testing. The test setup is shown in Figure 1.

**Figure 2:**
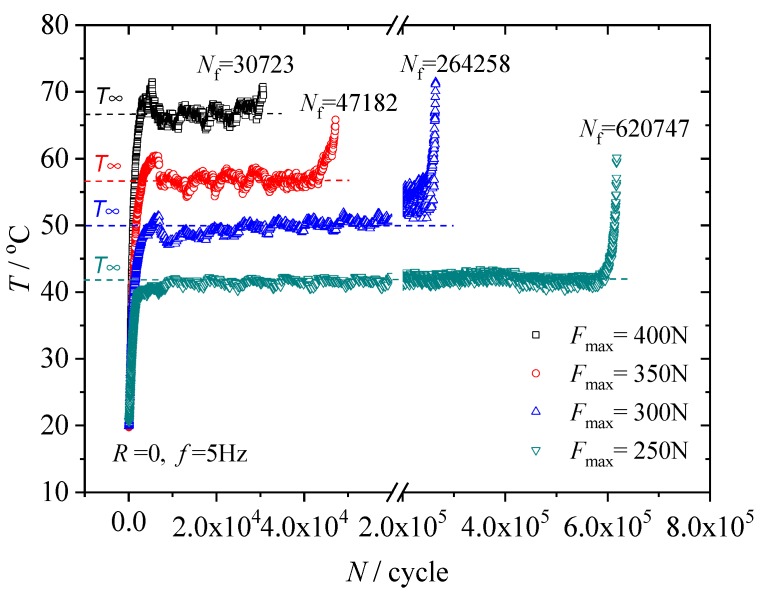
Surface temperature evolution of the specimen.

The maximum temperature values on the surface of the hourglass specimens were recorded during the fatigue-to-failure experiments by the thermal imaging camera. The discrepancies between duplicate measurements were small in all cases. Figure 2 shows the measured temperature evolution curves for four loading cases. It is obvious that the self-heating temperature increase was dependent on the loading conditions. The surface temperature rose rapidly during the first 3000–4000 cycles, and subsequently leveled off at a steady state temperature *T*_∞_, until it rose again sharply as failure approached. Fatigue failure of rubber material often occurs via crack propagation. At the moment of material fatigue failure, the crack growth rate in the material suddenly increases to infinity, that is to say, in a very short period of time, a large amount of energy is released due to the crack growth, which results in a sharp increase in the material temperature. Thus, the sharp increase in temperature can be regarded as a precursor to fatigue failure. It is also clear that the steady state temperature increases *θ*_∞_ (=*T*_∞_ − *T*_0_, where *T*_0_ is the initial temperature of the specimen) are different under various loading conditions, therefore *θ*_∞_ reflects the fatigue life in another manner, and can be considered a promising and alternative fatigue parameter.

## 3. Modeling and Discussions

### 3.1. Fatigue Damage Parameter Determination

The maximum principal strain $\varepsilon_{\max}$ was selected to be the fatigue parameter for constructing the S–N curve, as done in most studies in the literature [4,5,19]. To obtain the *ε*_max_ of the hourglass specimen under different force control fatigue loadings, a finite element simulation was employed, in which the material constitutive model was essential. As the material constitutive model determined from a single deformation mode experiment may not describe the mechanical response in other deformation modes, tests under three basic deformation modes are usually required in order to accurately establish the true constitutive model of rubber materials: simple tension (ST), equal-biaxial tension (ET) and planar tension (PT). Due to the incompressibility of the material, the ET of a rubber specimen creates a state of strain equivalent to pure compression, which can be accomplished by stretching the circumference of a circular specimen in 16 directions in a plane [20]. The PT test provides a state of pure shear in the specimen at a 45° angle to the stretching direction because of the perfectly lateral constrain, which can be easily performed on a universal tensile testing machine using a special fixture.

**Figure 3:**
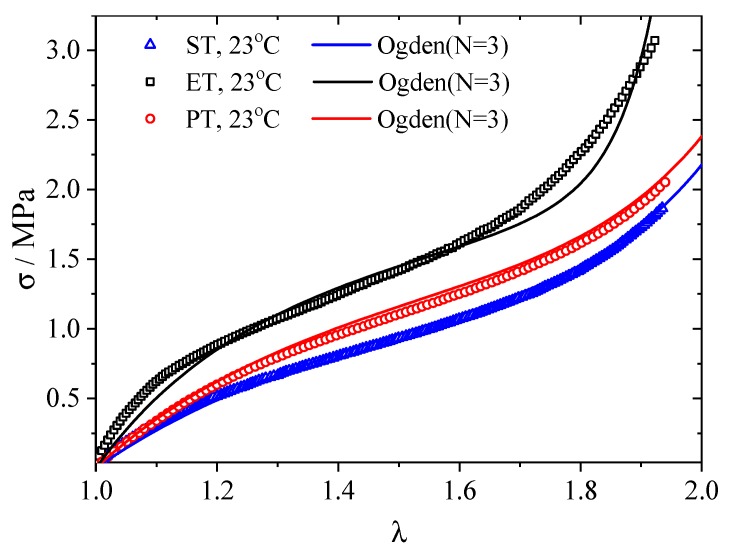
Stress–stretch curves for simple tension (ST), planar tension (PT) and equal-biaxial tension (ET) tests of rubber and their Ogden model fits.

The stress–stretch data of the filled rubber material in ST, ET and PT tests at 23 °C are shown in Figure 3. Such behavior is often modeled via hyperelastic idealization. By fitting a hyperelastic model to the test data, the constitutive model of the material can be determined. Many hyperelastic models have been developed. The relatively simple neo-Hookean and Mooney–Rivlin solids were the first hyperelastic models developed [21]; currently, the Arruda–Boyce model [22] and the Ogden model [23] are widely used to describe the strain state-dependent hyperelastic behavior, as depicted in Figure 3.

The Ogden strain density function [23], defined as

(1) $$W_{\mathrm{Ogden}}=\sum_{n=1}^{N}\frac{\mu_{n}}{\alpha_{n}}\left(\lambda_{1}^{\alpha_{n}}+\lambda_{2}^{\alpha_{n}}+\lambda_{3}^{\alpha_{n}}-3\right)$$

where $\lambda_{i}$ are the principal stretches,$\mu_{i}$ and *α_i_* are experimentally determined material constants, and *N* is the number of terms in the function, is considered one of the most successful functions in describing the hyperelasticity of rubber-like materials. The three-term model (*N* = 3) is used in this work to fit the experimental data, and the model parameters are identified as $\mu_{1}=1.9042$; $\mu_{2}=-1.924\times 10^{-10}$;$\mu_{3}=3.1850\times 10^{-4}$;$\alpha_{1}=1.0625$;$\alpha_{2}=-17.7$;$\alpha_{3}=12.3795$. The model fits are also shown by lines in Figure 3, indicating a satisfactory agreement with the tests. 

**Figure 4:**
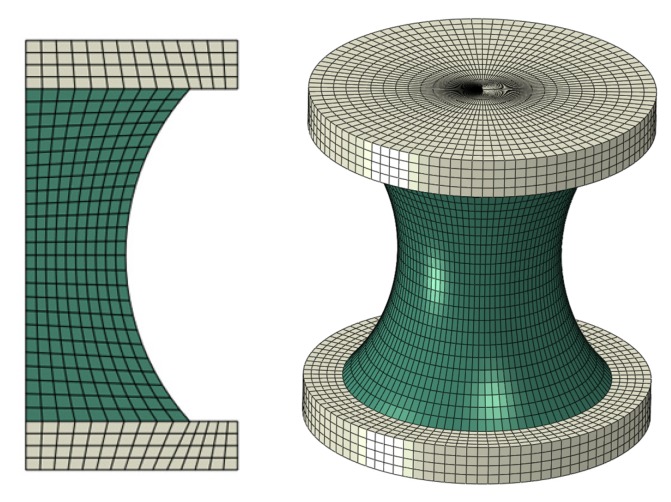
Axisymmetric geometric model of the hourglass rubber specimen (left: before rotation, right: after rotation).

**Figure 5:**
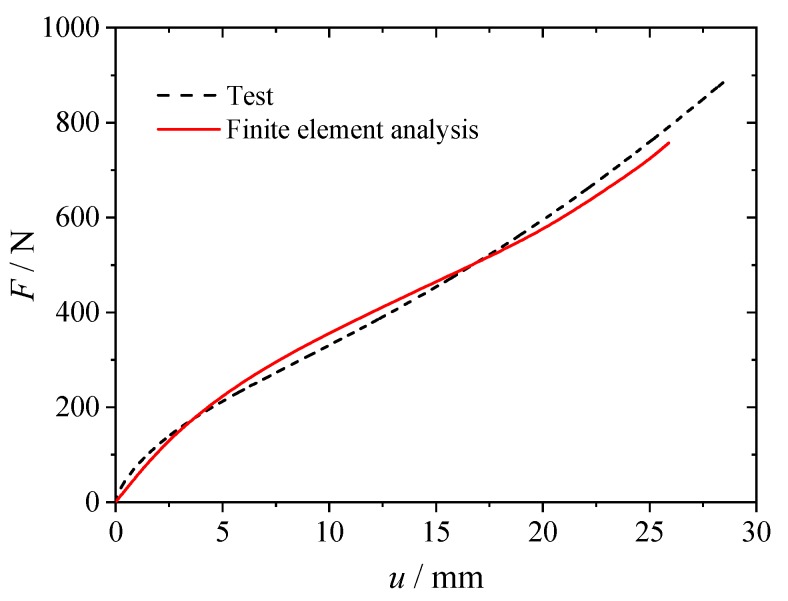
Tensile force-displacement curves of the hourglass specimen obtained from numerical analysis and lab tests.

A finite element model of the hourglass specimen used to calculate the $\varepsilon_{\max}$ is shown in Figure 4. The rubber part of the model was constructed by using four-node axisymmetric quadrilateral hybrid elements (CAX4H). The metal parts for clamping and loading at the top and bottom of the specimen used four-node axisymmetric quadrilateral elements (CAX4). In order to verify the three-term Ogden model described above, the uniaxial tensile behavior of the hourglass rubber specimen when stretched up to about 800N was numerically analyzed using the finite element model. The obtained force versus displacement relation of the specimen was compared with the lab test data, as shown in Figure 5. The good agreement between the numerical results and the experiment further indicates that the three-term Ogden model is an appropriate constitutive model for describing the hyperelastic behavior of the filled rubber material investigated in this study.

**Figure 6:**
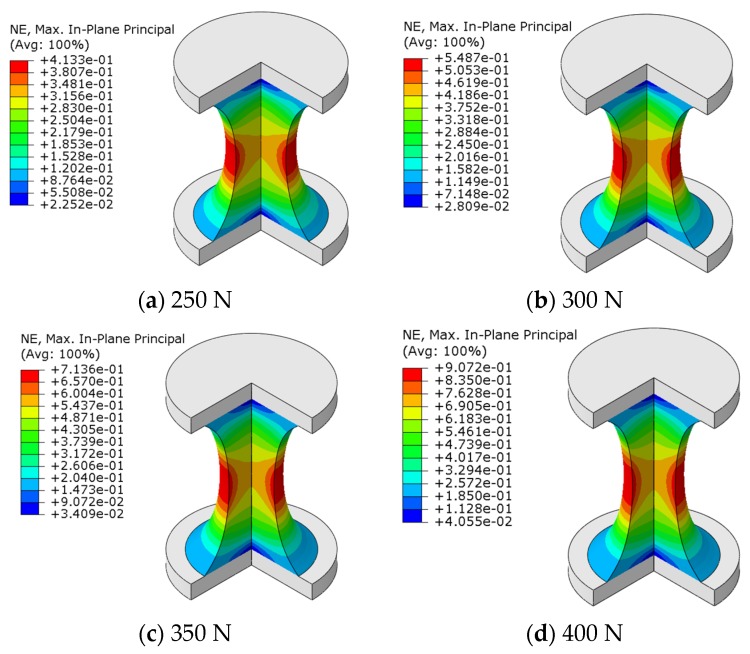
Maximum principal strain contours of the hourglass rubber specimen. (**a**) 250 N; (**b**) 300 N; (**c**) 350 N; (**d**) 400 N.

To get the maximum principal strain corresponding to the four loading cases in fatigue-to-failure experiments, finite element analyses with the three-term Ogden hyperelastic constitutive model were conducted. The bottom surface of the specimen was fixed, and four constant tensile forces (250 N, 300 N, 350 N, and 400 N) were applied to the top surface, respectively. Figure 6 shows the maximum principal strain contour plots of the specimen under the four designated tensile loads. The calculated maximum principal strains are listed in Table 1. 

### 3.2. Fatigue Life Assessment

**Figure 7:**
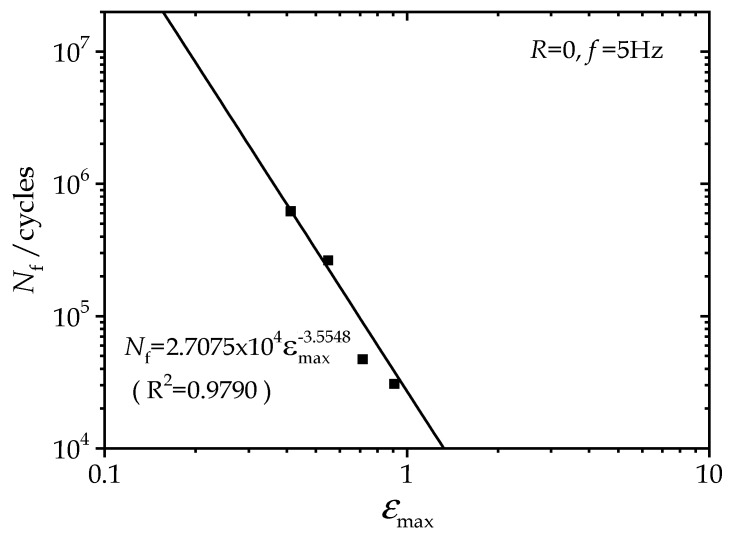
S–N curve of the hourglass rubber specimen.

In the fatigue-to-failure experiments, the fatigue lives for each loading case were recorded as seen in Figure 2. The maximum principal strains were obtained via finite element analysis, as described in the previous subsection. Considering maximum principal strain as the fatigue parameter, S–N curves can be built, as shown in Figure 7. 

Numerous studies demonstrate that the power law is an excellent model for relating the fatigue life and the maximum strain [4,5,19]. As depicted in Figure 7, the power law model describes the experimental S–N curve very well with Equation (2).

(2) $$N_{f}=k\varepsilon_{\max}^{n}=2.7075\times 10^{4}\varepsilon_{\max}^{-3.5548}$$

**Figure 8:**
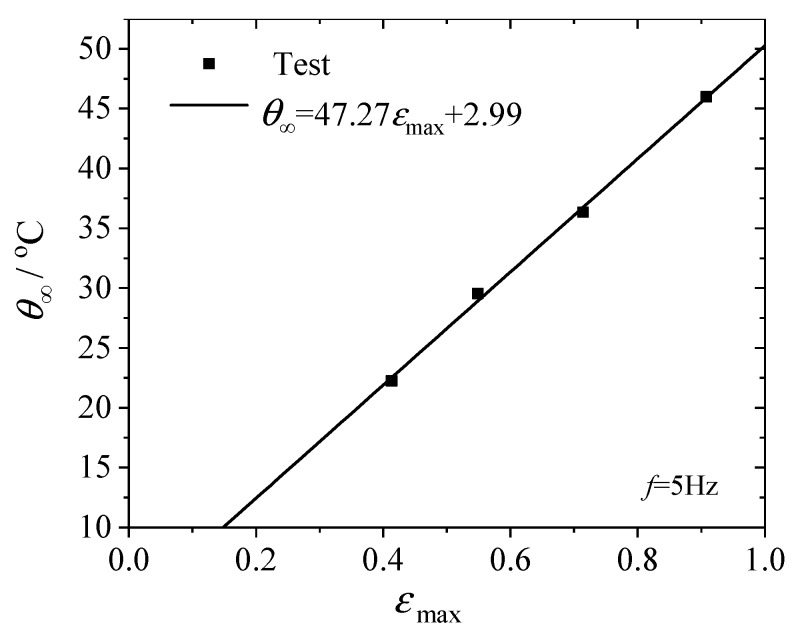
Steady state temperature increase of the hourglass specimen under fatigue with different $\varepsilon_{\max}$.

**Figure 9:**
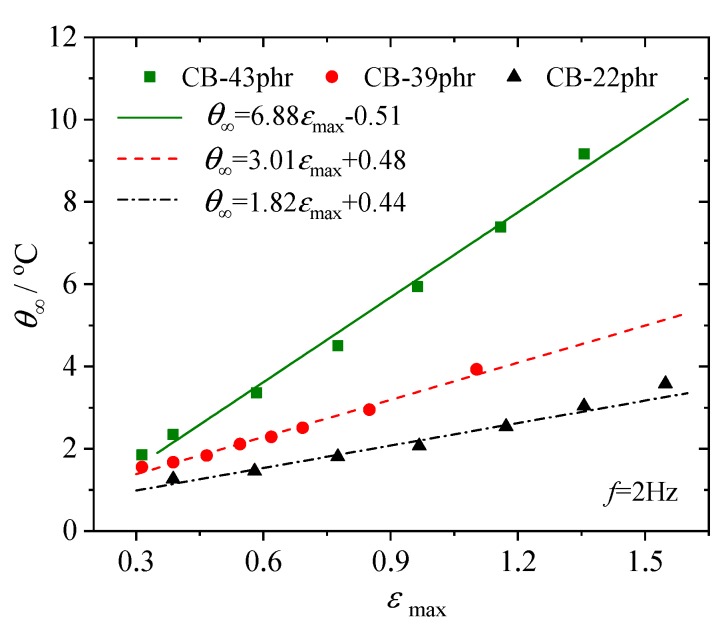
*θ*_∞_ vs. $\varepsilon_{\max}$ data for cyclic loaded filled rubber with various carbon black contents [14].

**Figure 10:**
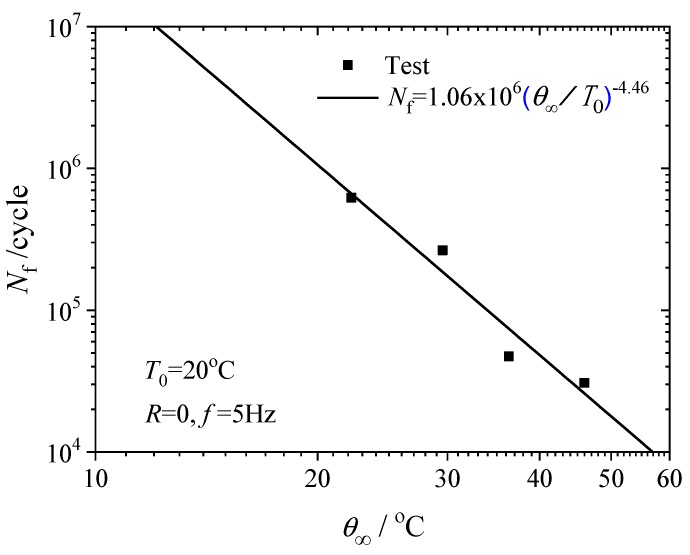
Fatigue lives vs. steady state temperature increases.

As mentioned in Section 2, the steady state temperature increase *θ*_∞_ can be considered as a promising and alternative fatigue parameter. We plotted the observed *θ*_∞_ with the corresponding maximum principal strain obtained by finite element simulation in Figure 8. Le Saux et al. [14] conducted similar heat build-up tests on several industrial rubber materials and discussed the correlation between the temperature rise and the principal maximum strain. In Figure 9, we redrew the *θ*_∞_ vs. $\varepsilon_{\max}$ data within the strain range from 0.3 to 1.5, approximately the same range as used in Figure 8, for carbon black filled natural rubber with different carbon black contents (22 phr, 39 phr and 43 phr). It is clear from Figure 8 and Figure 9 that the steady state temperature increase is approximately linearly proportional to the maximum strain in the considered range; therefore, the fatigue life would also be related to the steady temperature increase by a power law. We suggest a power law relation in the form of

(3) $$N_{f}=A{\left(\frac{\theta_{\infty }}{T_{0}}\right)}^{n}$$

where *T*_0_ is the initial temperature at the beginning of the fatigue test. Fitting the data in Figure 10 to the above equation with *T*_0_ = 20 °C yields the model parameters *A* and *n* in Equation (3); A = 1.06 × 10^6^, and *n* = −4.46. Equation (3) provides a promising criterion for the fatigue life prediction. As long as the steady state temperature increase at the point of the maximum strain is determined, the fatigue life of the structure can be predicted by the model.

Equation (3) bridges the fatigue life with the steady state temperature increase in the rubber material, rather than the maximum strain as expressed in Equation (2). The advantage is that the steady state temperature increase can be measured by thermal imaging in a few thousand cycles in fatigue tests, much fewer than the number of cycles to fatigue failure. Thus, the time cost for fatigue testing can be reduced and the characterization of the fatigue properties can be considerably accelerated by the method provided in this work. 

Based on the fatigue criterion suggested by Equation (3), thermomechanical coupling finite element simulation is expected to be the most promising method for fatigue life assessment. In such simulations, the loss energy density due to hysteresis in the dynamic viscoelasticity is considered as the heat resource in the deformed body. The steady temperature increase can be obtained by solving the heat equation with given initial boundary conditions [24], and the fatigue life of the rubber component can be subsequently determined. 

## 4. Conclusions

Fatigue tests and infrared thermal imaging measurements were carried out to correlate the fatigue life with the temperature increase induced by the hysteresis loss. Experiments show that the surface temperature of the specimen keeps its steady value for a prolonged period, which accounts for the majority of the fatigue life, and then sharply increases until the specimen ruptures. The sharp rise in temperature can be regarded as a precursor to fatigue failure. Moreover, the steady state temperature increase is linearly proportional to the maximum principal strain. By replacing the maximum principal strain, which is used as the fatigue life predictor in classic fatigue models, with the steady state temperature increase, a promising method for quickly evaluating the fatigue life of rubber structures is developed based on the power law model. Since only a couple of thousand cycles are required for the temperature to reach its steady state value during fatigue testing, which is less than one tenth of the fatigue life, the fatigue life of rubber components can be efficiently assessed by the steady state temperature increase.

## Figures and Tables

**Table 1:** The maximum principal strains of the hourglass specimen loaded with different maximum forces.

*F*_max_/N	250	300	350	400
$\varepsilon_{\max}$	0.4133	0.5487	0.7136	0.9072

## References

[1] Fan R.P., Meng G., Yang J., He C. (2009). Experimental study of the effect of viscoelastic damping materials on noise and vibration reduction within railway vehicles. J. Sound Vib..

[2] Brunac J.B., Gérardin O., Leblond J.B. (2009). On the heuristic extension of Haighs diagram for the fatigue of elastomers to arbitrary loadings. Int. J. Fatigue.

[3] Ayoub G., Nait-Abdelaziz M., Zairi F., Gloaguen J.M., Charrier P. (2012). Fatigue life prediction of rubber-like materials under multiaxial loading using a continuum damage mechanics approach: Effects of two-blocks loading and R ratio. Mech. Mater..

[4] Li Q., Zhao J., Zhao B. (2009). Fatigue life prediction of a rubber mount based on test of material properties and finite element analysis. Eng. Fail. Anal..

[5] Kim W., Lee H., Kim J., Koh S.K. (2004). Fatigue life estimation of an engine rubber mount. Int. J. Fatigue.

[6] Gent A.N., Lindley P.B., Thomas A.G. (1964). Cut growth and fatigue of rubbers. I. the relationship between cut growth and fatigue. J. Appl. Polym. Sci..

[7] Verron E., Andriyana A. (2008). Definition of a new predictor for multiaxial fatigue crack nucleation in rubber. J. Mech. Phys. Solids.

[8] Mars W.V. (2002). Cracking energy density as a predictor of fatigue life under multiaxial conditions. Rubber Chem. Technol..

[9] Doudard C., Calloch S., Cugy P., Galtier A., Hild F. (2005). A probabilistic two-scale model for high-cycle fatigue life predictions. Fatigue Fract. Eng. Mater. Struct..

[10] Rosa G.L., Risitano A. (2000). Thermographic methodology for rapid determination of the fatigue limit of materials and mechanical components. Int. J. Fatigue.

[11] Meneghetti G., Quaresimin M. (2011). Fatigue strength assessment of a short fiber composite based on the specific heat dissipation. Compos. Part B Eng..

[12] Jegou L., Marco Y., Le Saux V., Calloch S. (2013). Fast prediction of the Wöhler curve from heat build-up measurements on short fiber reinforced plastic. Int. J. Fatigue.

[13] Serrano L., Marco Y., Le Saux V., Robert G., Charrier P. (2017). Fast prediction of the fatigue behavior of short-fiber-reinforced thermoplastics based on heat build-up measurements: Application to heterogeneous cases. Contin. Mech. Thermodyn..

[14] Le Saux V., Marco Y., Calloch S., Doudard C., Charrier P. (2010). Fast evaluation of the fatigue lifetime of rubber-like materials based on a heat build-up protocol and micro-tomography measurements. Int. J. Fatigue.

[15] Luo W.B., Yin B.Y., Hu X.L., Zhou Z., Deng Y., Song K. (2018). Modeling of the heat build-up of carbon black filled rubber. Polym. Test..

[16] Zhi J., Wang S., Zhang M., Wang H., Lu H., Lin W., Qiao C., Hu C., Jia Y. (2019). Numerical analysis of the dependence of rubber hysteresis loss and heat generation on temperature and frequency. Mech. Time-Depend. Mat..

[17] Le Saux V., Marco Y., Calloch S., Charrier P., Taveau D. (2013). Heat build-up of rubber under cyclic loadings: Validation of an efficient demarch to predict the temperature fields. Rubber Chem. Technol..

[18] Katunin A. (2019). Criticality of the self-heating effect in polymers and polymer matrix composites during fatigue, and their application in non-destructive testing. Polymers.

[19] Mars W.V., Fatemi A. (2002). A literature survey on fatigue analysis approaches for rubber. Int. J. Fatigue.

[20] Li X.B., Wei Y.T. (2015). Classic strain energy functions and constitutive tests of rubber-like material. Rubber Chem. Technol..

[21] Mooney M. (1940). A theory of large elastic deformation. J. Appl. Phys..

[22] Arruda E.M., Boyce M.C. (1993). Three-dimensional constitutive model for the large stretch behavior of rubber elastic materials. J. Mech. Phys. Solids.

[23] Ogden R.W. (1972). Large deformation isotropic elasticity—On the correlation of theory and experiment for incompressible rubberlike solids. Proc. R. Soc. Lond. A Math. Phys. Sci..

[24] Li F.Z., Liu J., Yang H.B., Lu Y.L., Zhang L.Q. (2016). Numerical simulation and experimental verification of heat build-up for rubber compounds. Polymer.

